# Expanding the Zooarchaeology by Mass Spectrometry reference library for North American mammals: new collagen fingerprints from extinct and extant taxa

**DOI:** 10.1098/rsos.260792

**Published:** 2026-10-28

**Authors:** Mariya Antonosyan, Carli Peters, Eden Hill, Bryan Hockett, Robert S. Feranec, Madeline E. Mackie, Matthew Gibson, Dennis Jenkins, Torben C. Rick, Noel Amano, Patrick Roberts, Nicole Boivin

**Affiliations:** ^1^ Department of Coevolution of Land Use and Urbanization, Max Planck Institute for Geoanthropology, Jena, Germany; ^2^ Universidade do Algarve, Faro, Portugal; ^3^ Max Planck Institute for Geoanthropology, Jena, Germany; ^4^ Department of Geosciences, University of Tübingen, Tübingen, Germany; ^5^ Department of Anthropology, University of Nevada, USA; ^6^ New York State Museum, USA; ^7^ Department of Anthropology, Michigan State University, East Lansing, USA; ^8^ The Charleston Museum, USA; ^9^ Museum of Natural and Cultural History, University of Oregon, OR, USA; ^10^ Anthropology, National Museum of Natural History, Smithsonian Institution, Washington, DC, USA; ^11^ School of Social Science, University of Queensland Faculty of Arts, Saint Lucia, Queensland, Australia; ^12^ Griffith University, Queensland, Australia

**Keywords:** Zooarchaeology by Mass Spectrometry, zooarchaeology, palaeontology, North America

## Abstract

Robust zooarchaeological analysis of Pleistocene-Holocene faunal assemblages is fundamental for reconstructing past environments, species distributions, human subsistence and faunal turnovers in North America. However, faunal remains from archaeological and palaeontological sites are commonly highly fragmented or modified, often precluding morphological identification and limiting the information they can provide about faunal communities and their relationships with human populations and changing climates. Zooarchaeology by Mass Spectrometry (ZooMS) provides an effective biomolecular means of overcoming this limitation, enabling the identification of unidentifiable bone fragments, but its resolution depends on the availability of empirically validated reference datasets, which remain incomplete for North American fauna. Here, we expand the North American ZooMS reference library by characterizing and validating collagen peptide markers for 22 extant and extinct mammal taxa across four mammalian orders: Artiodactyla, Perissodactyla, Carnivora and Rodentia, integrating Matrix-Assisted Laser Desorption/Ionization Time-of-Flight Mass Spectrometry fingerprinting with Liquid Chromatography-Tandem Mass Spectrometry peptide sequencing to provide sequence-level validation of diagnostic peptide markers. The resulting markers enable robust family-level discrimination across a range of taxa with genus- or species-level identification possible for several groups. This expanded reference framework enhances the taxonomic resolution and applicability of ZooMS across North American faunal assemblages, supporting its use for advancing archaeological, palaeontological and conservation-related research on the continent.

## 1. Introduction

North America's terrestrial mammalian fauna is characterized by high taxonomic diversity [1,2] and a wide range of ecological adaptations spanning Arctic tundra, temperate forests, grasslands, arid deserts and tropical forests [3]. Many North American mammals are either endemic to the continent or represented by regionally restricted populations [4], giving the continent a unique faunal history with important implications for understanding long-term biogeographic and ecological change.

This distinctive fauna has undergone substantial changes in diversity and distribution throughout the Quaternary, driven by repeated glacial–interglacial climatic cycles and increasingly, from the terminal Pleistocene through the Holocene, by human impacts [5–7]. The most pronounced of these transformations was the Late Pleistocene megafaunal extinction, which resulted in the loss of approximately 38 mammalian genera from the North American continent through mechanisms that disproportionately affected large-bodied taxa [5,8]. Despite decades of research, the relative contributions of climate change, human activities and other potential impacts to their demise remain fiercely debated [6,9–13].

The drivers of more recent impacts on North American fauna are better understood, providing a clearer picture of the role of people in shaping ecosystems during the Holocene (e.g. [14–16]). In particular, the period of European colonial expansion that began in the late fifteenth century was marked by widespread landscape modification, large-scale resource extraction, the introduction of non-native species and the disruption of indigenous practices and ecological management [17–19], which together served to dramatically alter the health, distribution and resilience of native plant and animal populations. Currently, numerous North American mammal species are under threat, with 471 mammal taxa listed on the IUCN Red List of Threatened Species, including four Critically Endangered, 20 Endangered and 25 Vulnerable taxa. Many of North America's surviving large-bodied species face sustained conservation pressure, occupying climatic niches that differ markedly from those of the past 11 700 years [20]. Among North American mammals of conservation concern are the red wolf (*Canis rufus*), caribou (*Rangifer tarandus*) and Utah prairie dog (*Cynomys parvidens*) [21–25].

This long-term history of faunal change throughout the Pleistocene and Holocene provides an important context for understanding the ecological challenges facing North American mammals today and for informing effective conservation and management. Studying fossil and archaeological records to reconstruct the timing, magnitude and causes of faunal turnovers (e.g. [26–31]) provides critical data about climate change and its impacts, as well as ecological information about prior habitats and distributions that is critical for developing effective restoration strategies [32–39]. Improved understanding of extinctions and associated faunal dynamics can further reveal how the loss or decline of species reshaped ecological processes, such as nutrient cycling, trophic interactions and niche structure, and how these changes generated cascading effects and feedback loops that influenced long-term ecosystem trajectories [8,29].

Reliable zooarchaeological data are also critical for building effective reconstructions of human subsistence, behaviours, mobility and adaptation through time. Long-standing debates in the archaeological record of North America concern the extent of human exploitation of extinct megafauna [6,9–12,40], the relative contribution of large and small game to subsistence economies, the drivers of shifts in hunting intensity over time and the factors influencing prey selection [40–49].

These issues are particularly central to research on the palaeo-Indian period (approximately 13 500–8000 cal BP), during which early groups have often been viewed as specialized big-game hunters [50–52]. Increasing archaeological evidence, however, indicates that palaeo-Indian groups probably employed broader-spectrum subsistence strategies, involving diverse faunal and floral resources [45,46,53]. Understanding these strategies also requires examining how environmental changes—including warming temperatures, increasing monsoonal precipitation and an overall decrease in the abundance of large game across the landscape during the Holocene—affected the diets and prey choices of palaeo-Indian foragers [46,54]. Beyond terrestrial hunting, zooarchaeological evidence provides insights into the exploitation of aquatic resources, including marine mammals and fish, and their role in shaping human subsistence strategies and facilitating the dispersal of maritime peoples [55–59]. Zooarchaeology can also shed light on human relationships with animals beyond subsistence, particularly their importance in the production of critical perishable technologies, including clothing, tools, ornaments and weaponry. Secure taxonomic identification of worked bone and other artefacts, in the North American context, allows more precise evaluation of raw material selection strategies [60–65].

Despite their considerable interpretive potential, the value of archaeological faunal assemblages in North America is often constrained by their high degree of fragmentation. Such fragmentation results from taphonomic processes, human activities and post-depositional disturbance [41,66], limiting traditional zooarchaeological, palaeontological and palaeoecological analysis, leaving many specimens taxonomically unidentified and constraining reconstructions of past biodiversity, faunal exploitation and human–animal interactions. This problem is particularly pronounced for closely related taxa with similar skeletal morphology, where diagnostic features may be absent in fragmented remains. This includes large ungulates, such as bison (*Bison* spp.), elk (*Cervus canadensis*), moose (*Alces alces*) and musk ox (*Ovibos moschatus*), as well as medium-sized ungulates, such as bighorn sheep (*Ovis canadensis*), mountain goat (*Oreamnos americanus*), caribou (*R. tarandus*) and pronghorn (*Antilocapra americana*) [67–72].

Molecular approaches can help overcome the limitations imposed by severe bone fragmentation and other taphonomic impacts by enabling the retrieval of taxonomic information from bone fragments unidentifiable with morphological zooarchaeological analysis. One such method is Zooarchaeology by Mass Spectrometry (ZooMS), which allows taxonomic identification of archaeological bone fragments based on enzymatically digested collagen type I (COL1), the most abundant protein in bone [73]. In mammals, birds, reptiles and amphibians, COL1 is made up of two *α*1 chains and one *α*2 chain, encoded by the *COL1A1* and *COL1A2* genes. Taxon-specific nucleotide variation in these genes gives rise to differences in the amino acid sequences of COL1 among taxa. The basis of ZooMS is the generation of peptide mass fingerprints from tryptic digests of bone or other collagen-containing tissues using Matrix-Assisted Laser Desorption/Ionization Time-of-Flight mass spectrometry (MALDI-TOF-MS) [74]. This method of collagen fingerprinting can allow rapid and cost-effective taxonomic identification of highly fragmented bone, even in the absence of diagnostic morphological features [75,76]. ZooMS is accordingly a powerful tool to help reconstruct patterns of faunal diversity through time, capable of informing and supporting species conservation efforts [77–79], environmental reconstruction [80,81] and the analysis of human–faunal relationships [82–84].

ZooMS has been applied across a growing range of archaeological contexts in North America, demonstrating its value for investigating how technological and cultural innovations shaped human adaptation and for exploring the functional and symbolic relationships between humans and animals. ZooMS analysis of Late Pleistocene bone needles and worked hides has provided more detailed evidence for the emergence of tailored clothing in North America by identifying the species of their manufacture [61,62]. The method has also been applied to personal ornaments, bone points and other forms of material culture, revealing patterns of raw material selection, craft production and animal exploitation that are often obscured by morphological analysis alone [60,63]. Alongside these applications, considerable effort has focused on developing minimally destructive and non-destructive sampling approaches that preserve the physical integrity of rare, unique or culturally significant objects while enabling reliable taxonomic identification [63,64,85].

ZooMS has also proved highly effective for investigating the exploitation of marine mammals and fish in North America, substantially improving our understanding of resource use in coastal environments and the role of aquatic taxa in past subsistence economies [58,59,85–88]. Its application to legacy museum collections has also demonstrated its potential for reconstructing past human–animal interactions from previously unidentifiable archaeological material while maximizing the research value of existing collections [86,89]. Beyond archaeology, ZooMS has become an important tool in North American palaeoecology, contributing to studies of evolutionary history [90], Late Quaternary extinctions [91,92], demographic change [93], long-term ecological transformation [94], dietary reconstruction [95,96] and the historical spread of invasive species [97].

However, the accuracy of ZooMS identifications depends on the availability of reliable and well-characterized reference datasets for both extant and extinct taxa. Because identification relies on matching the peptide mass fingerprint of an unknown specimen to diagnostic markers established from taxonomically identified reference specimens, gaps in these datasets directly limit the taxonomic resolution and scope of ZooMS identifications. To date, such reference libraries, comprising established peptide markers against which unknown specimens can be compared, have been largely developed for Eurasian mammals [73,98], with more recent efforts extending to African [99,100], Australian [101,102], New Guinean [103] and South American [104] taxa. Progress has also been made in generating markers for non-mammalian taxa, including birds [105], reptiles [106], amphibians [107] and fish [108]. Coverage of North American mammals nonetheless remains relatively limited, particularly for native taxa and extinct Pleistocene species. Early studies established diagnostic collagen markers for several North American taxa and broader global taxa present in North America, including bison (*Bison* spp.), musk ox (*O. moschatus*), elk (*A. alces*), dog (*Canis lupus*) and red fox (*Vulpes vulpes*) [73]. Subsequent efforts have aimed at refining and enlarging this reference framework [75]. More targeted research has addressed specific zooarchaeologically relevant groups, including leporids, such as black-tailed jackrabbit (*Lepus californicus*), white-tailed jackrabbit (*L. townsendii*) and snowshoe hare (*L. americanus*) [60], as well as canids and felids, including grey fox (*Urocyon cinereoargenteus*), dire wolf (*Aenocyon dirus*), Canada lynx (*Lynx canadensis*) and saber-toothed cat (*Smilodon fatalis*) [61], particularly in the context of analyses of ancient bone artefact manufacture. Other studies have reported or predicted peptide mass markers for a limited number of additional North American taxa, such as white-tailed deer (*Odocoileus virginianus*), mule deer (*Odocoileus hemionus*), bobcat (*Lynx rufus*), the extinct cheetah-like cat (*Miracinonyx trumani*) and beaver (*Castor canadensis*) [109].

Importantly, however, many of these markers have been predicted from published COL1 gene sequences by reconstructing the corresponding collagen amino-acid sequences and identifying the expected tryptic peptides *in silico*, rather than being established through direct Liquid Chromatography-Tandem Mass Spectrometry (LC-MS/MS) measurements. For other taxa, reference markers remain entirely absent, highlighting the need both to validate existing predictions and to expand taxonomic coverage. Here, in order to improve the reliability and taxonomic coverage of the ZooMS reference dataset for North American terrestrial mammals, we validate previously predicted markers and establish new markers for extant and extinct taxa not previously represented. We validate previously predicted markers using LC-MS/MS, which determines the amino-acid sequences of the collagen peptides underlying the ZooMS markers and establishes whether differences in peptide mass observed by MALDI-TOF-MS reflect genuine amino acid substitutions rather than analytical artefacts. For both validation of predicted markers and the establishment of new ones, we integrate MALDI-TOF-MS fingerprints with LC-MS/MS peptide sequencing across multiple specimens per taxon, strengthening the taxonomic coverage and analytical robustness of the resulting ZooMS reference library while accounting for intraspecific variability. In total, we provide reference markers for 22 taxa across four major mammalian orders: Artiodactyla, Perissodactyla, Carnivora and Rodentia.

## 2. Material and methods

### 2.1. Material

In this study, we analysed faunal materials representing 22 taxa documented in North American archaeological and palaeontological contexts, including Late Pleistocene to Holocene records of the Great Basin [54,66], Great Plains [50,53] and Rocky Mountains [110,111]. The dataset encompasses a diverse range of both extant and extinct mammals, including ungulates, carnivores and rodents, selected to capture taxonomic breadth relevant to the analysis of archaeological assemblages across the continent. Ungulates are represented by pronghorn, caprines, cervids, camelids, equids and peccaries, including several extinct taxa, such as Harlan's muskox (*Bootherium bombifrons*), Yesterday's camel (*Camelops* sp.), large-headed llama (*Hemiauchenia* sp.), stag-moose (*Cervalces scotti*), American mountain deer (*Odocoileus lucasi*), stilt-legged horses (*Haringtonhippus* sp.), western horse (*Equus occidentalis)* and flat-headed peccary (*Platygonus compressus*). Carnivores include canids, felids and mustelids, notably extinct taxa, such as the American cheetah (*M. trumani*), while rodents are represented by beavers and porcupines. Multiple specimens were analysed for each taxon to account for possible intraspecific variability.

The analysed specimens were sourced from multiple United States institutions, including the Bureau of Land Management in Reno, Nevada, the University of Wyoming, the University of Oregon, the New York State Museum and the Charleston Museum. A full list of the analysed specimens, including information about taxonomy and provenance, is provided in table 1. For each specimen, approximately 30 mg of cortical bone was sampled for collagen extraction.

**Table 1.** List of analysed specimens. † Extinct taxa.

family	species	common name	source	ZooMS ID	LC-MS/MS ID
Antilocapridae	*Antilocapra americana*	Pronghorn	University of Wyoming	EHWY04	DA1142
Bovidae	*Oreamnos americanus*	Mountain goat	Bureau of Land Management: Reno	NAM-24	DA1330
	*Ovis canadensis*	Bighorn sheep	Bureau of Land Management: Reno	NAM-31	DA1335
	*Bootherium bombifrons* †	Harlan's muskox	The Charleston Museum	NAM-005	DA1249
Camelidae	*Camelops* sp. †	Yesterday's camel	Bureau of Land Management: Reno	NAM-41	DA1349
	*Hemiauchenia* sp. †	Large-headed llama	Bureau of Land Management: Reno	NAM-42	DA1350
Cervidae	*Odocoileus virginianus*	White-tailed deer	Bureau of Land Management: Reno	NAM-21	DA1337
	*Odocoileus hemionus*	Mule deer	Bureau of Land Management: Reno	NAM-22	DA1336
	*Odocoileus lucasi* †	American mountain deer	Bureau of Land Management: Reno	NAM-39	DA1353
	*Cervalces scotti* †	Stag-moose	New York State Museum NYSM VP-98	NAM-013	DA1250
Tayassuidae	*Platygonus compressus* †	Flat-headed peccary	New York State Museum NYSM VP-41	NAM-015	DA1251
Equidae	*Haringtonhippus* †	Stilt-legged horses	Bureau of Land Management: Reno	NAM-37	DA1346
	*Equus occidentalis* †	Western horse	University of Oregon	EHOR30	DA1155
Canidae	*Urocyon cinereoargenteus*	Gray fox	Bureau of Land Management: Reno	NAM-25	DA1340
	*Canis latrans*	Coyote	Bureau of Land Management: Reno	NAM-32	DA1332
	Vulpes velox	Swift fox	University of Wyoming	EHWY31	DA1154
Felidae	*Lynx rufus*	Bobcat	Bureau of Land Management: Reno	NAM-27	DA1342
	*Felis concolor*	Cougar	Bureau of Land Management: Reno	NAM-30	DA1334
	*Miracinonyx trumani* †	American cheetah	University of Wyoming	EHWY27	DA1150
Mustelidae	*Taxidea taxus*	North American badger	Bureau of Land Management: Reno	NAM-26	DA1341
Castoridae	*Castor canadensis*	North American beaver	Bureau of Land Management: Reno	NAM-23	DA1339
Erethizontidae	*Erethizon dorsatum*	North American tree porcupine	Bureau of Land Management: Reno,	NAM-20	DA1338

### 2.2. Collagen extraction

Collagen was extracted following previously published acid-insoluble ZooMS protocols [73,112] at the Max Planck Institute of Geoanthropology. The bone fragments were demineralized in 500 µl of 0.6 M hydrochloric acid (HCl) for 3 days. The samples were rinsed three times with 200 µl of 50 mM AmBic buffer (ammonium bicarbonate, NH_4_HCO_3_; pH 8). Then 100 µl of AmBic was added and the samples were gelatinized at 65°C for 1 hour. Samples were then briefly centrifuged at 9000 r.p.m. and 50 µl of supernatant was transferred to a fresh 1.5 ml microcentrifuge tube for subsequent enzyme digestion. The sample was incubated overnight (18 hours) with 1 µl of trypsin solution (0.4 µl µg^–1^; Pierce™ Trypsin Protease, Thermo Scientific) at 37°C, after which 1 µl of 5% trifluoroacetic acid (TFA) was added to stop enzymatic action. The peptides were purified using C18 ZipTip pipette tips (Pierce™ C18 Tips, Thermo Scientific).

### 2.3. Matrix-Assisted Laser Desorption/Ionization Time-of-Flight Mass Spectrometry

All digests were mixed with matrix solution (10 mg of *α*-cyano-4-hydroxycinnamic acid in 7 ml of 85% acetonitrile/0.1% TFA). A total of 3 µl of collagen extract was mixed with 3 µl of matrix solution. Then 1 µl of the sample was spotted in triplicate, each with a different calibrant/reference spot, on a 384-well Bruker MALDI ground steel target plate. The samples were run on a Bruker Autoflex Speed MALDI-TOF mass spectrometer (Bruker Daltonics) to produce spectra for taxonomic identification. We used an AutoXecute run in reflector mode (800–3700 *m/z*), with peak resolution greater than 5000, laser power fuzzy control ‘on’, initial laser power = 25, maximum laser power = 45, random walk and sum of shots = 800. A SNAP averaging algorithm was used to obtain monoisotopic masses (C: 4.9384, N: 1.3577, O: 1.4773, S: 0.0417, H: 7.7583). ZooMS screening was carried out at the Max Planck Institute of Geoanthropology.

### 2.4. Liquid Chromatography with Tandem Mass Spectrometry

A subsample of 20 µl from each collagen extract was dried down and analysed using LC-MS/MS at the Functional Genomics Centre Zurich (FGCZ). At FGCZ, mass spectrometry analysis was performed on an Orbitrap Exploris 480 mass spectrometer (Thermo Fisher Scientific) equipped with a Nanospray Flex Ion Source (Thermo Fisher Scientific) and coupled to an M-Class ultra-performance liquid chromatography (UPLC) instrument (Waters). Solvent composition in the two channels was 0.1% formic acid for channel A and 0.1% formic acid, 99.9% acetonitrile for channel B. Column temperature was 50°C. For each sample, 5 µl of peptides were loaded on a commercial nanoEase MZ Symmetry C18 trap column (100 Å, 5 µm, 180 µm × 20 mm, Waters) followed by a nanoEase MZ C18 HSS T3 column (100 Å, 1.8 µm, 75 µm × 250 mm, Waters). The peptides were eluted at a flow rate of 300 nl min^–1^. After a 3 min initial hold at 5% B, a gradient from 5 to 22% B in 40 min and 22 to 32% B in an additional 5 mins was applied. The column was cleaned after the run by increasing to 95% B and holding 95% B for 10 min before re-establishing the loading condition for another 10 min.

The mass spectrometer was operated in data-dependent mode with a maximum cycle time of 3 s, funnel radio frequency (RF) level at 40%, heated capillary temperature at 275°C and advanced peak determination on. Full-scan MS spectra (350–1200 *m/z*) were acquired at a resolution of 120 000 at 200 *m/z* after accumulation to a target value of 300% or for a maximum injection time of 50 ms. Precursors with an intensity above 5000 were selected for MS/MS. Ions were isolated using a quadrupole mass filter with a 1.2 *m/z* isolation window and fragmented by higher-energy collisional dissociation (HCD) using a normalized collision energy of 30%. HCD spectra were acquired at a resolution of 30 000, with a maximum injection time of 119 ms. The automatic gain control was set to 100%. Charge state screening was enabled such that singly, unassigned and higher than seven charge states were rejected. Precursor masses previously selected for MS/MS measurement were excluded from further selection for 20 s and the exclusion window was set at 10 ppm. The samples were acquired using internal lock mass calibration on *m/z* 371.1012 and 445.1200.

### 2.5. Identification and confirmation of biomarkers

For the identification and confirmation of collagen peptide markers, MALDI spectra were visualized via mMass software v. 5.5.058 [113]. Before peak identification, spectra were pre-processed in mMass by applying baseline correction with a precision of 15 and a relative offset of 25, followed by smoothing using the Savitzky–Golay method with a window size of 0.3 *m/z* and two smoothing cycles. Monoisotopic peak lists were generated by peak picking, employing a signal-to-noise threshold of 3.5 and the spectra were subsequently deisotoped. Each spectrum was then evaluated visually for the presence of 12 standard marker peaks. Candidate peptide markers were selected based on comparisons to a list of published collagen markers [60,61,73,98,109]. The selected candidate peptide markers were confirmed through analysis of the LC-MS/MS data using Byonic v. 3.2.0 (Protein Metrics Inc.; [114]) following a step-by-step approach [59]. A detailed description of the search settings, databases used and quality controls for confident identification can be found in the supplementary material.

All raw data files, databases and results were uploaded to ProteomeXchange with identifier PXD076625. MALDI-TOF-MS spectra are available on Mendeley Data: doi:10.17632/fnvnb7wttk.1.

## 3. Results

All samples yielded MALDI-TOF-MS spectra suitable for the identification of COL1 markers. Between 3840 and 8470 peptide spectrum matches were identified in the non-error-tolerant search of the LC-MS/MS data. Collagen was identified as the main protein component in all LC-MS/MS samples and common contaminants (reported in the supplementary material) were not detected in high quantities. Together, these analyses provide confirmed collagen peptide markers for 22 North American mammal taxa across four major mammalian orders: Artiodactyla, Perissodactyla, Carnivora and Rodentia (table 2; corresponding peptide sequences are reported in supplementary material, table S1). Some markers require cautious interpretation because their *m/z* values overlap among different taxa or peptide markers, as indicated in table 2.

**Table 2.** Newly established and/or empirically confirmed ZooMS peptide markers. Peptide markers in bold can be used to differentiate between taxa in the same family. Peptide markers underlined and in italics should be used with caution since these *m/z* values overlap between different taxa/peptide markers. Peptide markers in parentheses represent the same marker with an additional oxidation. Naming of peptide markers follows Brown *et al.* [115].

family	species	COL1*α*1 508–519 (P1)	COL1*α*2 978–990 (A)	COL1*α*2 375–386	COL1*α*2 449	COL1*α*2 484–498 (B)	COL1*α*2 502–519 (C)	COL1*α*2 889–906	COL1*α*2 292–309 (P2)	COL1*α*2 793–813 (D)	COL1*α*2 454–483 (E)	COL1*α*1 586–618 (F)	COL1*α*2 757–789 (G)
Antilocapridae	pronghorn (*A. americana*)	1105	1180 (1196)	*1182 (1198)*	1287/ 3324^c^ (3340^c^)	1427	1580	-	1648	2131	2792	2883 (2899)	3017^b^ (3033)
Bovidae	mountain goat (*O. americanus*)	1105	1180 (1196)	*1154(1170)*	1287	1427	1580	1560	1648	2131	2792	2883 (2899)	3017^b^ (3033)
	bighorn sheep (*O. canadensis*)	1105	1180 (1196)	*1154(1170)*	1287	1427	1580	1560	1648	2131	2792	2883 (2899)	3017^b^ (3033)
	Harlan's muskox (*B. bombifrons*)	1105	*1192(1208)****^a^***	*1182(1198)*	1287	1427	1580	**1532**	1648	2131	2792	**2853 (2869)**	3017^b^ (3033)
Camelidae	Yesterday's camel (*Camelops* sp.)	1105	1205 (1221)	*1182 (1198)*	**1273****^d^****/3311^cd^**	1453	1550	*1566*	1634	2131	2820	2883 (2899)	3017^b^ (3033)
	large-headed llama (*Hemiauchenia* sp.)	1105	1205 (1221)	*1182 (1198)*	**1287****^d^****/3324****^c^****(3340^cd^)**	1453	1550	*1566*	1634	2131	2820	2883 (2899)	3017^b^ (3033)
Cervidae	white-tailed deer *(O. virginianus*)	1105	1180 (1196)	*1182 (1198)*	1287/ 3324^c^ (3340^c^)	1427	1580	1532	1648	2131	2792	2883 (2899)	3043 (3059)
	mule deer (*O. hemionus*)	1105	1180 (1196)	*1182 (1198)*	1287/ 3324^c^ (3340^c^)	1427	1580	1532	1648	2131	2792	2883 (2899)	3043 (3059)
	American mountain deer (*O. lucasi*)	1105	1180 (1196)	*1182 (1198)*	1287/ 3324^c^ (3340^c^)	1427	1580	1532	1648	2131	2792	2883 (2899)	3043 (3059)
	stag-moose (*C. scotti*)	1105	1180 (1196)	*1182 (1198)*	1287	1427	**1550**	1532	1648	2131	2792	2883 (2899)	**3017****^b^****(3033)**
Equidae	stilt-legged horse (*Haringtonhippus* sp.)	1105	*1182 (1198)*	*1182 (1198)*	1287/ 3324^c^ (3340^c^)	1427	1550	1604	1649	2145	2820	2883 (2899)	2983 (2999)
	western horse (*E. occidentalis*)	1105	*1182 (1198)*	*1182 (1198)*	1287/ 3324^c^ (3340^c^)	1427	1550	1604	1649	2145	2820	2883 (2899)	2983 (2999)
Tayassuidae	flat-headed peccary (*P. compressus*)	1105	*1154 (1170)*	*1154 (1170)*	1287	1453	1550	1532	1647	2145	2820	2883 (2899)	3017^b^ (3033)
Canidae	grey fox (*U. cinereoargenteus*)	1105	1210 (1226)	1214 (1230)	1257	1453	*1566*	1548	1593 (1609)	2131	2820	2853 (2869)	2983 (2999)
	coyote (*C. latrans*)	1105	1210 (1226)	1214 (1230)	1257	1453	*1566*	**1576**	1593 (1609)	2131	2820	2853 (2869)	2983 (2999)
	swift fox (*V. velox*)	1105	1210 (1226)	1214 (1230)	1257	1453	*1566*	1548	1593 (1609)	2131	2820	2853 (2869)	2983 (2999)
Felidae	bobcat (*L. rufus*)	1105	1207 (1223)	1214 (1230)	1257/ 3342^c^ (3358^c^)	1453	*1566*	1588	1593 (1609)	2163	2820	2853 (2869)	2983 (2999)
	cougar (*F. concolor*)	1105	1207 (1223)	1214 (1230)	1257/ 3342^c^ (3358^c^)	1453	*1566*	1588	1593 (1609)	2163	2820	2853 (2869)	2983 (2999)
	American cheetah (*M. trumani*)	1105	1207 (1223)	1214 (1230)	**1287****^d^**	1453	*1566*	1588	1593 (1609)	2163	2820	2853 (2869)	2983 (2999)
Mustelidae	North American badger (*T. taxus*)	1105	*1182 (1198)*	1214 (1230)	1271	1453	*1566*	1548	1593 (1609)	2147	2820	2853 (2869)	2983 (2999)
Castoridae	North American beaver (*C. canadensis*)	1105	*1177 (1193)**^a^*	*1182 (1198)*	1239	1427	1593	1576	1596	2129	2808	2883 (2899)	2983 (2999)
Erethizonidae	North American tree porcupine (*E. dorsatum*)	1105	1161 (*1177*)	-	1287	1453	1594	1604	1637	2143 (2159)	2862	2869 (2885)	2983 (2999)

a A different peptide sequence but with a corresponding mass has been observed in the MS/MS data of some (or all) analysed peptides. This marker should thus be used in combination with other peptide markers to make secure identifications.

b Another peptide with a peak at *m/z* 3017 is conserved across bovids [99].

c Same peptide but with a missed cleavage.

d Only observed in LC-MS/MS data.

The newly characterized markers differentiate all analysed families and provide additional taxonomic resolution within several families. Within artiodactyls, diagnostic marker combinations separate Bovidae, Antilocapridae, Cervidae, Camelidae, Tayassuidae and Equidae. Finer resolution is achieved within several of these families, including Cervidae (e.g. *Odocoileus* deer from stag-moose) and Camelidae (Yesterday's camel from large-headed llama). By contrast, Equidae cannot be resolved below family level, with stilt-legged horse and western horse displaying identical sets of markers. Among carnivores, the newly characterized markers distinguish the Felidae, Canidae and Mustelidae families, though genus- or species-level discrimination remains limited to selected taxa (e.g. coyote from swift fox) and closely related species, such as bobcat and cougar remain indistinguishable. Finally, the study expands the ZooMS reference library for rodents by characterizing diagnostic collagen fingerprints for North American beaver and North American tree porcupine. The sections below describe the taxonomic resolution of the ZooMS markers in more detail within each family.

### 3.1. Bovidae and Antilocapridae Zooarchaeology by Mass Spectrometry markers

Harlan's muskox (*B. bombifrons*) can be differentiated from the other bovids and antilocaprids in our study based on peptide markers COL1*α*2 978–990 observed at *m/z* 1192/1208, COL1*α*2 375–386 at *m/z* 1182 (1198), COL1*α*2 859–906 at *m/z* 1532 and COL1*α*1 586–618 at *m/z* 2853 (2869) (figure 1). However, its suite of peptide markers is identical to those of aurochs (*Bos primigenius*) and bison (*Bison bison*). Pronghorn (*A. americana*) can be differentiated from mountain goat (*O. americanus*) and bighorn sheep (*O. canadensis*) based on peptide marker COL1*α*2 375–386 (with peaks at *m/z* 1182/1198 for *pronghorn*). Interestingly, this peptide marker is shared between pronghorn, (domestic) sheep (*Ovis aries*) and argali (*Ovis ammon*) [73,109], while mountain goat and bighorn sheep show a different fingerprint.

**Figure 1.**
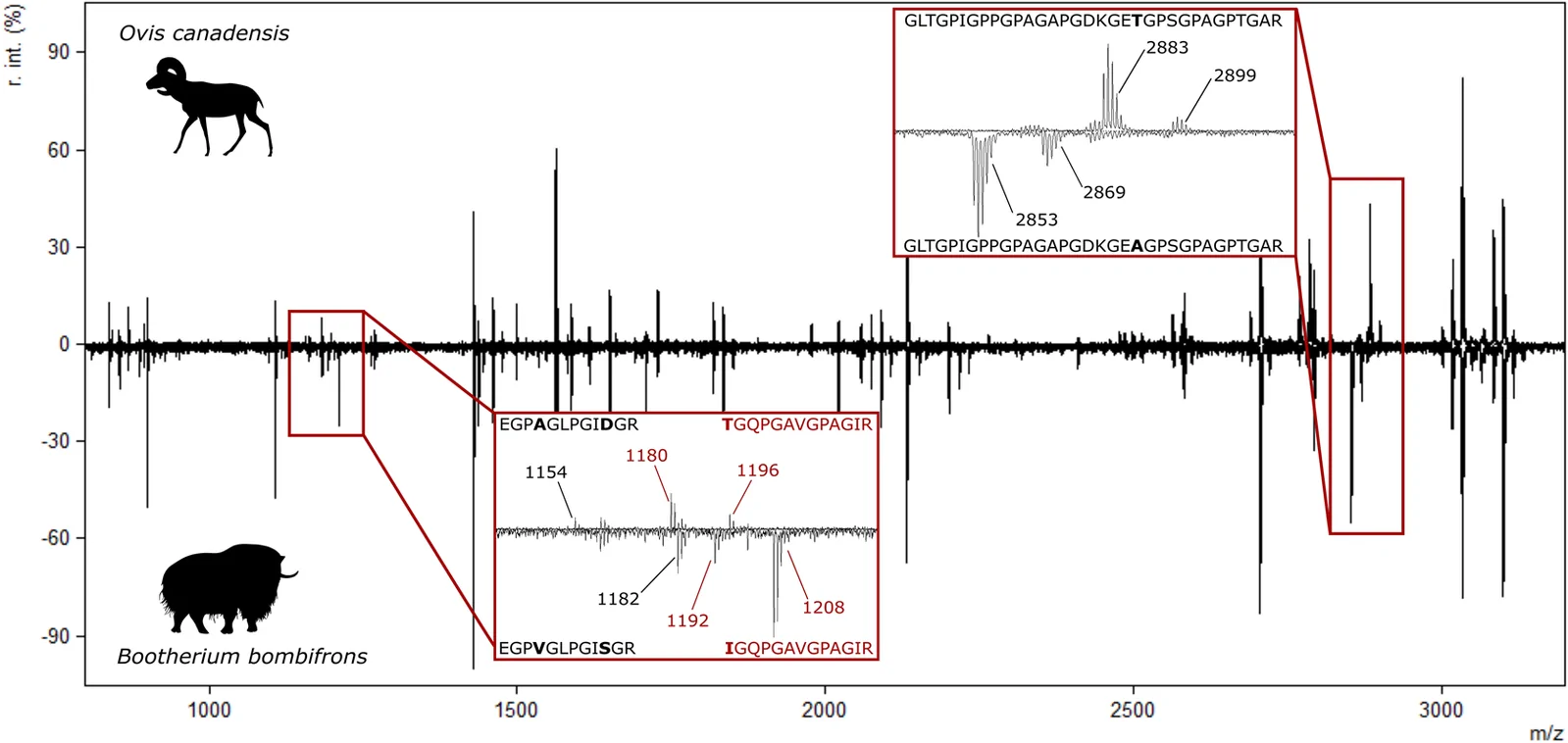
ZooMS spectra of bighorn sheep (*O. canadensis*) and Harlan´s muskox (*B. bombifrons*), highlighting the peptide markers that can be used to differentiate between them. The corresponding sequence information is indicated.

### 3.2. Cervidae Zooarchaeology by Mass Spectrometry markers

White-tailed deer (*O. virginianus*), mule deer (*O. hemionus*) and the extinct American mountain deer (*O. lucasi*) can be differentiated from other North American cervids based on the peptide marker COL1*α*2 757–789. This marker appears at *m/z* 3043 and 3059 for *Odocoileus* (figure 2). However, at the same time, we did not observe peptide marker differences within the *Odocoileus* genus, contradicting previous ZooMS reference data for mule deer [98]. Our dataset differs from this previous report at peptide marker COL1*α*2 502–519, which we observed at *m/z* 1580, but which was reported at *m/z* 1593. The collagen fingerprint of the extinct stag moose (*C. scotti*) is distinct from *Odocoileus* deer based on markers COL1*α*2 502–519 and COL1*α*2 757–789, producing peaks at *m/z* 1550 and 3017/3033, respectively. Notably, previously published LC-MS/MS data indicate that an additional peptide, conserved across all analysed bovid species, is detected at *m/z* 3017 [99]. Therefore, this marker can only be used reliably when both a peak at *m/z* 3017 and *m/z* 3033 are visible in the MALDI spectrum.

**Figure 2.**
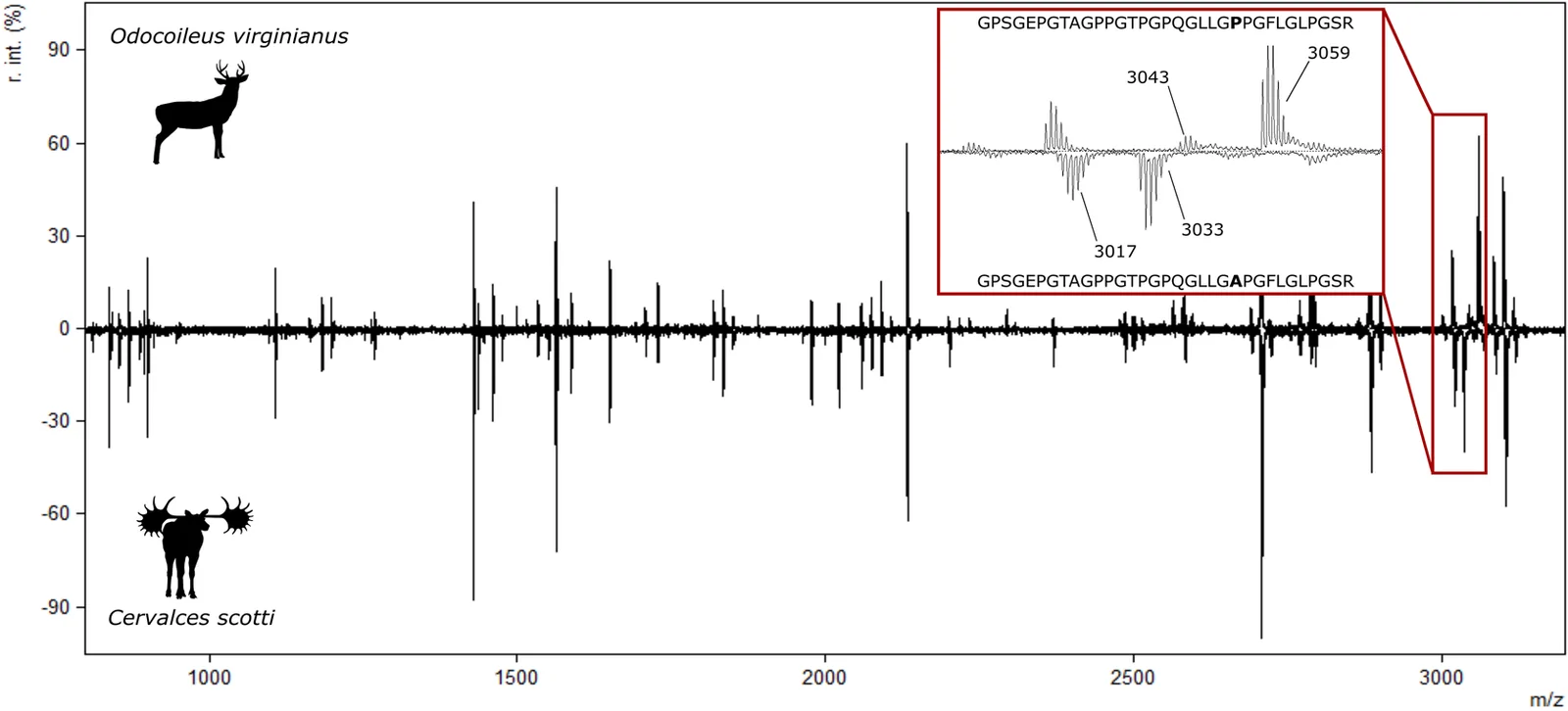
ZooMS spectra of white-tailed deer (*O. virginianus*) and stag moose (*C. scotti*) highlighting the peptide marker that can be used to differentiate between them. The corresponding sequence information is indicated.

### 3.3. Camelidae and Tayassuidae Zooarchaeology by Mass Spectrometry markers

It is possible to distinguish Camelidae from other artiodactyls through a combination of markers COL1*α*2 978–990, observed at *m/z* 1205 (1221); COL1*α*2 484–498, at *m/z* 1453; COL1*α*2 889–906, at *m/z* 1566 and COL1*α*2 292–309 at *m/z* 1634. At the same time, Yesterday´s camel (*Camelops* sp.) and large-headed llama (*Hemiauchenia* sp.) can be distinguished with ZooMS using peptide marker COL1*α*2 449. This marker appears at *m/z* 1273/3311 for Yesterday´s camel and *m/z* 1287/3324/3340 for large-headed llama (figure 3), although this peptide marker is only visible in the MALDI spectra of large-headed llama and should thus only be used to make positive identifications.

**Figure 3.**
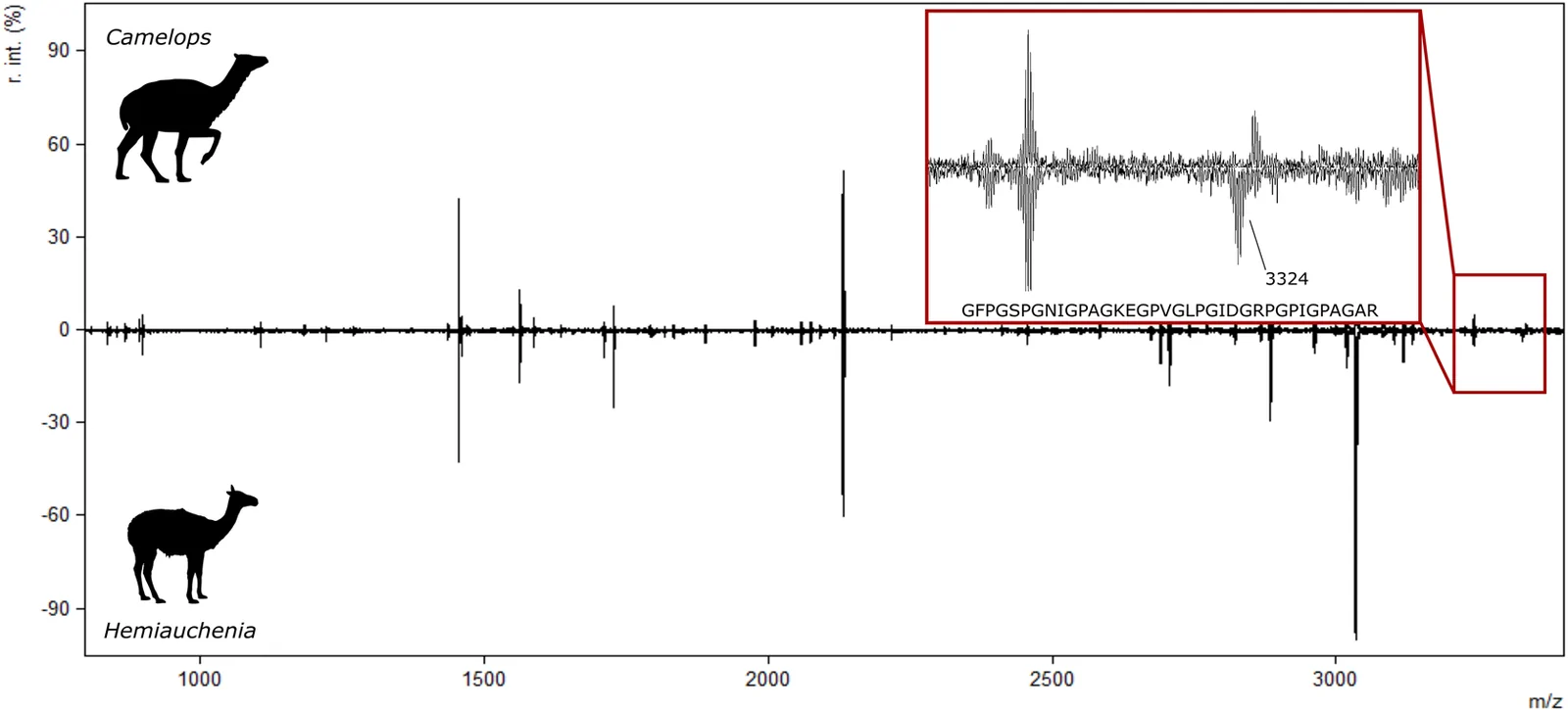
ZooMS spectra of Yesterday's camel (*Camelops* sp.) and large-headed llama (*Hemiauchenia* sp.), highlighting the peptide marker that can be used to differentiate between them. The corresponding sequence information is indicated.

North American camelids can be differentiated from vicuña (*Lama vicugna*) based on the peptide marker COL1*α*1 586–618, which is at *m/z* 2845/2861 in vicuña [73] compared with *m/z* 2883/2899 in North American camelids. Compared with extant dromedaries and camels (*Camelus* sp.) and the extinct Yukon giant camel (*Paracamelus* sp.), Yesterday's camel and large-headed llama show a difference at peptide marker COL1*α*2 757–789 (*m/z* 2975/2991 and *m/z* 3017/3033, respectively), which confirms previous observations by Buckley *et al.* [116] that these taxa can be differentiated with ZooMS.

Here, we report for the first time a collagen fingerprint for the flat-headed peccary (*P. compressus*) within the family Tayassuidae. A unique combination of peptide markers, COL1*α*2 978–990 at *m/z* 1154/1170; COL1*α*2 484–498 at *m/z* 1453; COL1*α*2 292–309 at *m/z* 1647; and COL1*α*2 793–813 at *m/z* 2145, allows its secure identification. The closest comparative taxon currently represented in the ZooMS record is the Chacoan peccary (*Catagonus wagneri*), a South American tayassuid for which only predicted peptide markers are available [109]. The predicted peptide markers of Chacoan peccary [109] reveal one difference at peptide marker COL1*α*2 793–813 compared with the flat-headed peccary (*m/z* 2131 and *m/z* 2145, respectively).

### 3.4. Equidae zooarchaeology by mass spectrometry markers

Members of the family Equidae can be distinguished from other North American ungulates based on two diagnostic collagen peptide markers: COL1*α*2 978–990, observed at *m/z* 1182/1198 and COL1*α*2 757–789, observed at *m/z* 2983/2999.4. Here, we report two extinct *Equus* taxa: stilt-legged horses (*Haringtonhippus* sp.) and the western horse (*E. occidentalis*). These two groups cannot be differentiated with ZooMS as they share an identical set of peptide markers. These two horse species also cannot be differentiated from other taxa in the Equidae family for which ZooMS markers were previously reported and/or predicted [73,75,98,109].

### 3.5. Felidae Zooarchaeology by Mass Spectrometry markers

All previously reported extant members of the family Felidae share an identical set of ZooMS markers, with the exception of the genus *Panthera*. For *Panthera*, peptide marker COL1*α*2 793–816 is observed at *m/z* 2147, whereas for other reported felids, *Felis* and *Lynx*, it is observed at *m/z* 2163 [61,73,98]. We expand and validate this dataset by generating ZooMS markers for three species: the bobcat (*L. rufus*), the cougar (*Puma concolor*) and the extinct jaguarundi (*Puma yagouaroundi*). We report that bobcat and cougar cannot be distinguished from one another using ZooMS. In addition, both share an identical collagen marker set with members of the genus *Felis*. However, all three can be separated from the genus *Panthera* based on the diagnostic COL1*α*2 793–816 marker.

The extinct American cheetah (*M. trumani*) differs from the other felids in our study at peptide marker COL1*α*2 449, at *m/z* 1287. However, this peptide marker was only identified in the MS/MS dataset. Peptide markers for the bobcat [98], cougar and American cheetah [61] were published previously, but are further expanded in this study. For the latter two, our data also validate previous observations that were made based solely on MALDI-TOF-MS data through association with sequence information obtained with LC-MS/MS. It is also noteworthy that collagen markers for the extinct genus of sabre-toothed cat (*Homotherium* sp.) have been predicted from genetic sequence data but have not yet been confirmed using MALDI-TOF-MS [109]. Based on these sequence-derived predictions, the peptide peak for COL1*α*2 484–498 is expected at *m/z* 1427 and COL1*α*2 793–816 at *m/z* 2161. These values differ from those observed in other felids, highlighting the need for empirical MALDI validation to confirm the ZooMS profile of this taxon.

### 3.6. Canidae and Mustelidae Zooarchaeology by Mass Spectrometry markers

Canids could generally be distinguished from other carnivores by the combination of peptides COL1*α*2 978–990, observed at *m/z* 1210/1226, COL1*α*2 484–498 at *m/z* 1453 (*m/z* 1437 for red fox: *V. vulpes*), COL1*α*2 793–816 at *m/z* 2131 and COL1*α*2 757–789 at *m/z* 2869/2983. Peptide markers for all three canid taxa included in our study were previously reported [61,98], but these reports were based solely on MALDI observations or predicted masses. Here, we validate these findings by combining both MALDI data and collagen sequence information and expanding the number of available peptide markers for these taxa. The grey fox (*U. cinereoargenteus*) and swift fox (*Vulpes velox*) share an identical set of markers. At the same time, these can be distinguished from the coyote (*Canis latrans*) using peptide marker COL1*α*2 889–906, which is expressed at *m/z* 1548 and *m/z* 1576, respectively (figure 4). This follows previous findings that this peptide marker can be used to distinguish between *Canis* and *Vulpes* [73].

**Figure 4.**
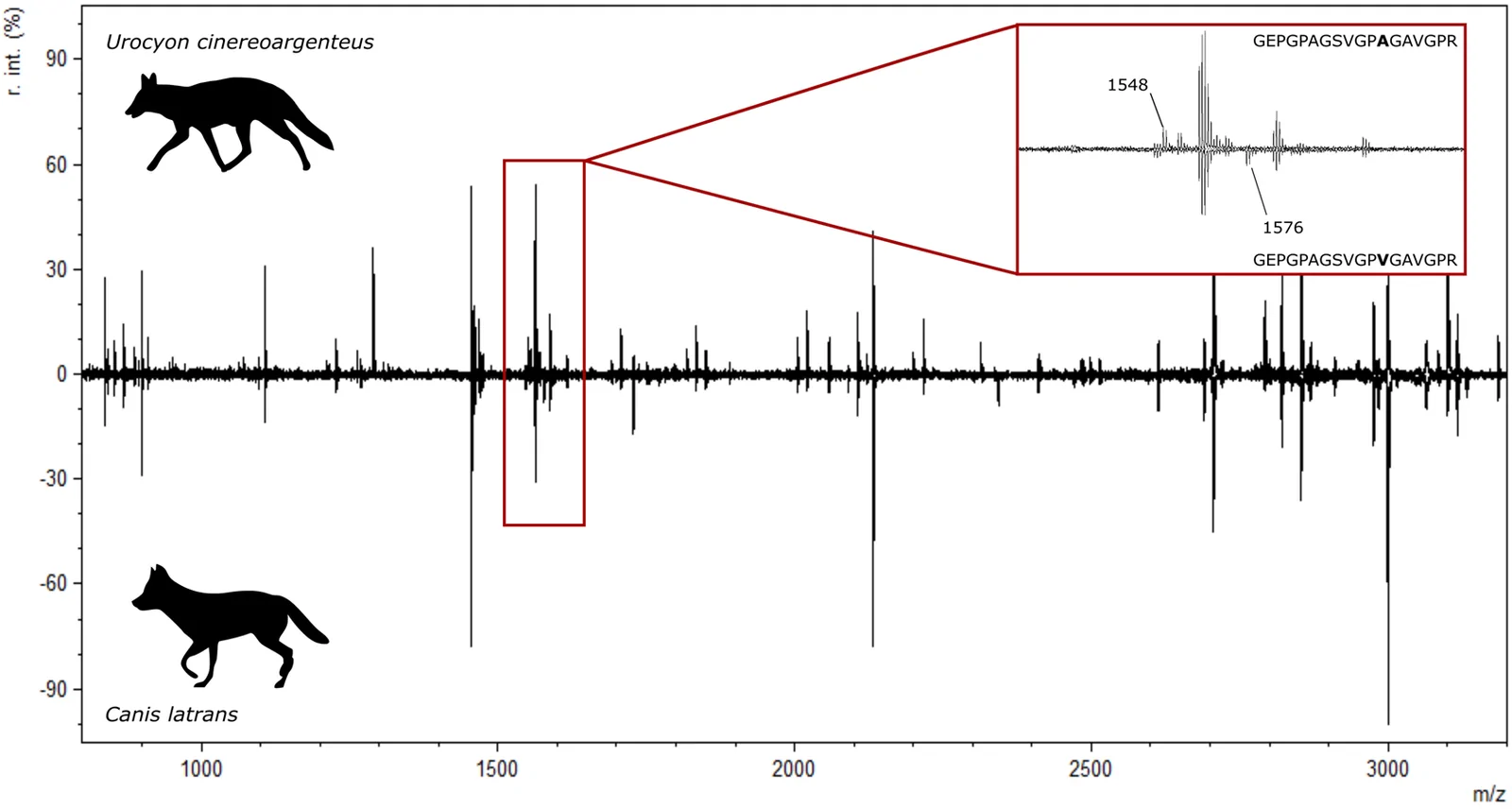
ZooMS spectra of the grey fox (*U. cinereoargenteus*) and the coyote (*C. latrans*), highlighting the peptide marker that can be used to differentiate between them. The corresponding sequence information is indicated.

We report the collagen fingerprint of the North American badger (*Taxidea taxus*), allowing its differentiation from other carnivores through a combination of peptides COL1*α*2 978–990 (*m/z* 1182/1198), COL1*α*2 449 (*m/z* 1271), COL1*α*2 889–906 (*m/z* 1548), COL1*α*2 375–386 (*m/z* 1214/1230) and COL1ɑ2 757–789 (*m/z* 2983/2999). Notably, the collagen fingerprint of the North American badger differs from that of the European badger (*Meles meles*) at peptide markers COL1*α*2 978–990 and COL1*α*2 757–789 [73,98,109]. Further, the North American badger expresses a different peptide marker at COL1*α*2 978–990 (*m/z* 1182/1198) compared with all other mustelid genera for which ZooMS reference data are currently available (*Mustela* sp., *Gulo* sp., *Lutra* sp.*, Enhydra* sp. and *Meles* sp.).

### 3.7. Rodentia Zooarchaeology by Mass Spectrometry markers

We report collagen fingerprints for the North American beaver (*C. canadensis*) and the North American porcupine (*Erethizon dorsatum*). Our dataset confirms the previously predicted set of peptide markers for the North American beaver [109] and demonstrates that it can be distinguished from the European beaver (*Castor fibre*). This distinction is based on the COL1*α*2 292–309 peptide marker, which appears at *m/z* 1596.8 in the North American beaver.

The North American porcupine differs clearly from the North American beaver by exhibiting distinct *m/z* values for seven peptide markers, allowing straightforward separation of these two large North American rodents. We also report clear differences from the closest comparative taxon currently represented in the ZooMS record, Old World porcupines (*Hystrix* sp.) [109]. In particular, peptides COL1*α*2 292–309, COL1*α*2 793–816 and COL1*α*1 586–618 in North American porcupines are observed at *m/z* 1637, 2143/2159 and 2869/2885, respectively.

## 4. Discussion

This study substantially expands and strengthens the ZooMS reference framework for North American terrestrial mammals, providing empirically validated markers for 22 extant and extinct taxa. The expanded reference dataset enables discrimination among a range of closely related ungulates, carnivores and rodents, including several extinct taxa, while also identifying groups for which collagen fingerprints remain insufficiently diagnostic. Together, these findings provide a more robust basis for applying ZooMS to North American archaeological and palaeontological assemblages.

The value of this increased resolution extends beyond taxonomic identification. Closely related North American taxa of similar body size are often particularly difficult to distinguish in fragmented assemblages, yet may differ substantially in their distributions, habitat preferences, ecological roles and relationships with human populations. Distinguishing among these taxa, while also establishing where ZooMS cannot provide such resolution, is therefore important for maximizing the information recovered from fragmented assemblages and identifying cases where morphological, contextual or additional biomolecular evidence remains necessary. Below, we consider the taxonomic resolution achieved across the major groups analysed in our study, as well as its limitations, and explore the implications for archaeological and palaeontological research, including reconstructions of human–animal interactions, past species distributions and environmental change.

### 4.1. Distinguishing ungulates using Zooarchaeology by Mass Spectrometry

All analysed species within the Bovidae, Antilocapridae and Cervidae families share six collagen peptide markers, reflecting the limited capacity of ZooMS to discriminate among closely related mammalian taxa. We report peptide markers for three bovid species: the extinct Harlan's muskox, the extant mountain goat and the bighorn sheep. A distinct peptide combination distinguishes Harlan's muskox from most other bovids and from cervids. However, Harlan's muskox shares an identical marker set with bison. These taxa also significantly overlapped in size and shared much of their range during the Late Pleistocene [117,118]. Since ZooMS alone cannot distinguish between them, identifications should be supported by morphology, archaeological context and/or additional biomolecular data.

Interestingly, the ZooMS markers identified here for Harlan's muskox do differ from those reported for its sister taxon, the extant musk ox (*O. moschatus*) [73], despite phylogenetic analyses based on mitogenome data supporting a close relationship between the two species, with their divergence time estimated at *ca* 250 kyr BP [119,120]. However, the previously reported extant musk ox markers were predicted from genetic data and not validated using LC-MS/MS, highlighting the need for empirical confirmation of these assignments.

Our dataset enables the differentiation of pronghorn from similarly sized artiodactyls, including mountain goat and bighorn sheep, and further allows all three to be distinguished from deer in the *Odocoileus* genus. However, we did not detect collagen marker differences among the three analysed *Odocoileus* species (*O. virginianus, O. hemionus* and *O. lucasi*), making their separation using ZooMS currently impossible. Notably, previously published data reported a unique marker for mule deer (*O. hemionus*) based solely on MALDI data [98]. This discrepancy may reflect intraspecific variability [121], though this cannot be confirmed owing to the absence of peptide sequence data in the original study.

Among cervids, we identify markers that allow the extinct stag moose to be distinguished from bovids and antilocaprids. However, its collagen fingerprint is identical to that of wapiti (*C. canadensis*) and moose (*A. alces*) and it thus cannot be uniquely identified with ZooMS. It also cannot be differentiated from saiga (*Saiga tatarica*), which was present in North America during the Pleistocene but is now extirpated from the continent [122]. Importantly, there are several other now-extinct North American bovid, antilocaprid and cervid species for which ZooMS reference data are not available (e.g. four-horned pronghorn (*Stockoceros*), dwarf pronghorn (*Capromeryx*), four-pronged pronghorn (*Tetrameryx*) and shrubox (*Euceratherium*)). We thus cannot rule out the possibility that these species share overlapping peptide markers.

Yesterday's camel and the large-headed llama were megafauna species that became extinct at the end of the Pleistocene [123–126]. They display distinct peptide marker sets that clearly separate them from other artiodactyls, consistent with their evolutionary divergence from these groups. In addition, they differ from each other by a single peptide marker, potentially allowing their taxonomic differentiation where collagen preservation is sufficient. ZooMS data are also available for the extinct Yukon giant camel (*Paracamelus*), allowing its separation from Yesterday's camel and the large-headed llama [116]. By contrast, no ZooMS data are currently available for the extinct camelid genus *Palaeolama*, which also inhabited North America during the Pleistocene.

Among tayassuids, the extinct flat-headed peccary is the first species in its genus (*Platygonus*) to be analysed with ZooMS. We report a unique set of collagen peptide markers that enables its secure identification and distinction from the closest comparative taxon for which ZooMS peptide markers are currently available, the Chacoan peccary (*C. wagneri*) [109]. Another tayassuid species present in North America during the Pleistocene, the long-nosed peccary (*Mylohyus nasutus*), has not yet been characterized using ZooMS. The extant collared peccary (*Pecari tajacu*) is also currently absent from the ZooMS reference dataset. However, there is no confirmed Pleistocene record of the collared peccary from North America, and its Pleistocene distribution appears to have been restricted much farther south [127]. Until reference markers are generated for these tayassuid taxa, specimens identified as flat-headed peccary based on collagen fingerprinting should be interpreted with caution in regions or time periods where long-nosed peccary and/or collared peccary may also have been present.

We observe no ZooMS marker differences between equids. The extinct stilt-legged horse and the western horse display an identical set of peptide markers. These two horse species also cannot be differentiated from other taxa in the Equidae family, as current ZooMS markers based on trypsin digestion lack resolution below the genus level. Despite these limitations, the improved taxonomic differentiation achieved for several of the species analysed here allows more precise reconstruction of diachronic changes in subsistence strategies. In regions such as the Great Basin and California, this could clarify whether the observed Late Holocene increase in artiodactyl remains reflects shifts in resource availability, population pressure, technological change or decisions shaped by nutritional ecology and optimal foraging strategies [41,54]. Furthermore, the ability to distinguish pronghorn (typically associated with open grassland environments [128]) from mountain goat and bighorn sheep, which preferentially inhabit steep and rocky terrain [129,130], is valuable for reconstructing habitat use.

The identification of extinct taxa, such as Harlan's muskox, stag-moose, flat-headed peccary, Yesterday's camel and the large-headed llama also demonstrates the potential of ZooMS to contribute to resolving long-standing debates surrounding Late Pleistocene megafauna extinctions in North America [6,7,9–12,131,132]. Improved identification of fragmented remains can increase the number of specimens available for reconstructing the distribution and chronology of these taxa, providing a stronger basis for assessing patterns of population decline and extinction. In addition, refined taxonomic identifications can also help elucidate changes in ecological niche and community organization following Pleistocene extinctions [8].

### 4.2. Distinguishing carnivores using Zooarchaeology by Mass Spectrometry

We report ZooMS markers for three felid species: bobcat, cougar and the extinct jaguarundi. Bobcat and cougar share an identical set of collagen peptide markers, making their distinction using ZooMS impossible. This marker set is also shared with most other felids for which peptide markers have been published previously [61,73,98,109], including other North American taxa, such as Canadian lynx (*L. canadensis*) and jaguarundi (*P. yagouaroundi*). Nonetheless, these felids can be distinguished by ZooMS from members of the genera *Panthera* and *Homotherium*, including extinct North American taxa, such as the American lion (*Panthera atrox*) and sabre-toothed cat (*Homotherium serum*).

Jaguarundi differs from the other felids in our study at peptide marker COL1*α*2 449. However, this marker was detected only in the LC-MS/MS dataset and can thus only be used to exclude jaguarundi as a potential identification. Furthermore, since this peptide marker is newly identified here, we do not know its counterpart in other felids previously analysed with ZooMS and cannot exclude the possibility that it is shared with other felids.

We also validate peptide markers for three canid species: grey fox, coyote and swift fox [61,98]. We confirm that grey fox and swift fox can be distinguished from coyotes using ZooMS. However, Arctic fox (*Vulpes lagopus*) and dhole (*Cuon alpinus*), which are also native to North America, cannot be differentiated from grey fox and swift fox. All four taxa can be distinguished from red fox (*V. vulpes*), which displays a unique collagen fingerprint.

We also report collagen peptide markers for the North American badger, enabling its differentiation from several other mustelids present in North America, including martens (*Martes* sp.), polecats and weasels (*Mustela* sp.) and the wolverine (*Gulo gulo*). It is important to note, however, that ZooMS reference markers are not yet available for several additional North American mustelids, including the American mink (*Neogale vison*), North American river otter (*Lontra canadensis*) and greater grison (*Galictis vittata*). Therefore, while the reported markers allow separation from currently represented comparative taxa, further reference development is required to fully resolve all regional mustelid diversity.

Evidence for carnivore hunting in the North American archaeological record is scarce compared with the extensive record of large herbivore exploitation, potentially reflecting human prey selection, low carnivore population densities and carnivore avoidance behaviours. Nonetheless, carnivore use has been documented at Bonneville Estates Rockshelter, where remains include bobcat and a large canid (either dire wolf or grey wolf), as well as a large felid (either American cheetah or cougar) [31]. ZooMS offers a means to expand evidence for carnivore exploitation, particularly when applied to large assemblages dominated by highly fragmented and morphologically unidentifiable remains. ZooMS identification of carnivores in archaeological and fossil sites can also help reconstruct the biogeography and foraging behaviour of past carnivore populations. For example, Mychajliw *et al.* [93] identified a short-faced bear (*Arctodus simus*) on California's San Miguel Island using ZooMS, a finding that raised questions about how this extinct species came to be present on an offshore island. Application of the markers developed here could similarly refine our understanding of the past distributions of other North American carnivores.

Beyond detecting carnivores in the archaeological record, ZooMS can help clarify how they were used, including the contribution of fur-bearing carnivores to clothing production. At the La Prele Mammoth site, ZooMS analysis has provided evidence for tailored garment production, involving bone needles and pelts from fur-bearing carnivores [61], directly linking carnivore exploitation to clothing manufacture. Comparable bone tools and worked osseous artefacts are widely curated in North American museum collections, many of which were excavated decades ago and lack secure taxonomic assignments. Improved ZooMS reference data can expand the research potential of these legacy collections and reveal the contribution of carnivores to artefact manufacture [133]. Protein preservation in North American specimens has already been shown to be suitable for ZooMS, enabling reanalysis of previously unidentifiable specimens [89]. Moreover, minimally destructive sampling techniques allow researchers to revisit culturally significant, rare or unique materials while largely preserving their physical integrity [63,134–138]. The suitability of these approaches, however, depends on factors including collagen preservation and sample condition, and more invasive sampling may be required where collagen recovery is insufficient. By providing taxonomic information through minimally invasive sampling where preservation permits, ZooMS can facilitate biomolecular research while supporting the conservation and responsible management of museum collections.

### 4.3. Distinguishing rodents using Zooarchaeology by Mass Spectrometry

Rodents are represented by two taxa in our dataset: the North American beaver and the North American porcupine. These two taxa have unique collagen fingerprints, and we confirm the previously predicted set of peptide markers for North American beaver [109], supporting the conclusion that it can be differentiated from European beaver (*C. fibre*) using ZooMS. The North American porcupine is the first species of its genus (*Erethizon*) for which ZooMS markers have been developed. Its closest relative for which ZooMS markers are available is the Old-World porcupine (*Hystrix*) [109]. These two taxa differ at several peptide markers.

Other large rodent taxa reported in North American contexts include several species of marmot (e.g. groundhog (*Marmota monax*) and hoary marmot (*Marmota caligata*)) and the extinct genera *Castoroides*, *Hydrochoerus* and *Neochoerus*. The predicted set of peptide markers for groundhog [109] indicates that it can be differentiated from both the North American beaver and the North American porcupine. No ZooMS data is currently available for the three extinct large rodent genera. Moreover, numerous smaller-bodied rodent taxa were not included in the present study and are likewise almost entirely absent from the existing ZooMS reference database, with the exception of some arvicoline rodents [139].

The development of new ZooMS markers improves not only the study of human–animal interactions but also the reconstruction of past environments. When applied, for example, to faunal assemblages from natural traps and cave deposits, ZooMS can enable more accurate species identification in contexts that are not strongly shaped by human activity. This can refine reconstructions of past species distributions, range shifts, refugia and mobility patterns, especially when combined with stable isotope analysis [92,93]. Rodents, owing to their narrow ecological tolerances and habitat specificity, are particularly informative in this regard, as they provide sensitive indicators of environmental change [140–143]. Better taxonomic resolution of rodent remains can therefore improve palaeoenvironmental reconstructions and enable a more detailed assessment of local climatic conditions. Future studies integrating ZooMS with stable isotope analysis of small mammals could provide finer-scale reconstructions of past environmental variability.

North American beavers are critical ecosystem engineers in wetland environments, and there is considerable interest in their ecological recovery following widespread population declines caused by overhunting during the fur trade [144,145]. Yet we know relatively little about the biogeography and distribution of beaver populations during the Pleistocene and earlier [146]. ZooMS can help expand our understanding of the deep history of North American beaver populations, the timing of extinctions among Pleistocene beaver species, such as the giant beaver, and the long-term context for their modern ecological recovery [144–146].

## 5. Conclusions

This study expands the North American ZooMS reference library by providing validated collagen peptide markers for 22 extant and extinct mammalian taxa across four major orders: Artiodactyla, Perissodactyla, Carnivora and Rodentia. By integrating MALDI-TOF-MS collagen fingerprinting with LC-MS/MS peptide sequencing, we establish a robust reference framework that improves the taxonomic resolution of ZooMS analyses for North American archaeological and palaeontological assemblages. The resulting improvements in taxonomic identification can strengthen reconstructions of past human–animal interactions, biodiversity changes and ecological dynamics, while also providing a foundation for broader applications in climate research and contemporary conservation efforts.

Despite the substantial expansion of the North American ZooMS reference dataset presented here, several limitations remain. The primary constraint is the limited taxonomic resolution among closely related (and sometimes ecologically overlapping) groups. COL1 is highly conserved and many diagnostic peaks are thus shared between related groups. This issue is clearest in Bovidae, Antilocapridae and Cervidae, where significant overlap in peptide markers limits the resolution of resulting identifications (e.g. Harlan's muskox sharing an identical marker set with bison; stag-moose being indistinguishable from elk and moose; and the absence of diagnostic markers within the *Odocoileus* genus). This underscores the need to consider species distributions and to integrate ZooMS results with zooarchaeological evidence in order to achieve robust taxonomic interpretations. Further, we demonstrate that peptide fingerprints generated following tryptic digestion do not provide sufficient resolution to differentiate horse species from other members of the Equidae family. However, previous research by Paladugu *et al.* [147] reported a novel ZooMS peptide marker generated using chymotrypsin digestion that reliably distinguishes between horses and donkeys. This demonstrates that enzyme choice can increase taxonomic resolution within equids and raises the possibility that an alternative enzyme to trypsin could also enable differentiation among extinct North American horses. However, testing this possibility was beyond the scope of the present study.

Secondly, despite the expanded taxonomic coverage provided here, many taxa are still absent from the North American ZooMS reference dataset. This includes regional ungulate, carnivore and rodent taxa (e.g. shrub ox, dwarf pronghorn, long-nosed peccary and wolverine) as well as smaller-bodied terrestrial mammals. Furthermore, non-mammalian terrestrial taxa, including birds, reptiles and amphibians, are still largely absent from existing North American ZooMS reference libraries, with only limited data currently available for select bird taxa [105].

Future research is therefore necessary to further expand ZooMS reference libraries to include taxonomic groups that are currently under-represented and to validate peptide markers using LC-MS/MS analysis. In addition, further methodological development may help overcome limits to taxonomic resolution. Testing such approaches across key North American taxa is a promising direction for improving ZooMS taxonomic resolution and ultimately enabling species-level identifications for a wider range of taxa. The expanded and empirically validated reference framework presented here provides a strong foundation for these future developments and for the wider application of ZooMS to North American faunal assemblages.

## Supplementary Material



## Data Availability

All raw data files, databases and results were uploaded to ProteomeXchange with identifier PXD076625. MALDI-TOF-MS spectra are available on Mendeley Data [148]. Supplementary material is available online [149].
