# Supplementary material for: Expanding the Zooarchaeology by Mass Spectrometry reference library for North American mammals: new collagen fingerprints from extinct and extant taxa
Source: R Soc Open Sci. 2026 Oct 28;13(10):260792. doi: 10.1098/rsos.260792 (PMC13714420; doi:10.1098/rsos.260792)
Supplement: Supplementary file 1 [file rsos.260792.zip › rsos.260792/rsos260792_si_001.docx]

**Expanding the ZooMS reference library for North American mammals: New collagen fingerprints from extinct and extant taxa**

**Supplementary Information**

**Supplementary Methods**

Identification and confirmation of peptide biomarkers

For the identification and confirmation of collagen peptide markers for ZooMS, the LC-MS/MS data was searched in a stepwise approach consisting of multiple searches.

First, MS/MS data was searched against a database consisting of the COL1α1 and COL1α2 sequences of *Bison bison bison* (XP_010841089.1; XP_010838069.1), *Bos taurus* (XP_001029211.1; XP776945.1), *Capra hircus* (XP_017920382.1; XP_005678993.1), *Ovis aries* (XP_027830506.1; XP_004007775.1), *Camelus bactrianus* (XP_010947400.1; XP_010970903.1), *Camelus dromedarius* (XP_031325489.1; XP_010977966.1), *Vicugna pacos* (XP_006218715.1; XP_006207690.1), *Odocoileus virginianus texanus* (XP_020769669.1; XP_020769440.1), *Loxodonta africana* (XP_010592644.1; XP_010585560.1), *Mammut americanum* (P0C2W8; P85154), *Equus caballus* (XP_023508478.1; XP_001492989.1), *Sus scrofa* (XP_020922812.1; XP_001230584.1), *Mylodon darwinii* (C0HJP3; C0HJP4), *Dasypus novemcinctus* (XP_023445022.1; XP_004470764.1), *Canis lupus familiaris* (XP_001003090.1; XP_001003187.1), *Vulpes vulpes* (XP_025841655.1; XP_025859557.1), *Puma concolor* (only COLα2: XP_025785714.1), *Puma yagouaroundi* (XP_040353137.1; XP_040328586.1), *Panthera leo* (XP_042771858.1; XP_042771858.1), *Lynx canadensis* (XP_030152285.1; XP_030163826.1), *Hyaena hyaena* (XP_039083754.1; XP_039092171.1), *Ursus arctos horribilis* (XP_026368914.1; XP_026361636.1), *Ursus maritimus* (XP_040496745.1; XP_008684476.1), *Castor canadensis* (XP_020026973.1; XP_020019043.1), and common contaminants. The parameter settings used were: cleavage sites fully specific C-term arginine (R) and lysine (K); 3 missed cleavages allowed; mass changes - 6 common, 0 rare; oxidation on K, methionine (M), and proline (P); deamidation on asparagine (N) and glutamine (Q); false discovery rate (FDR) 2%. Peptide sequences (with at least 3 spectral matches and with a PEP2D score <0.01) corresponding to manually identified candidate peptide markers were recorded.

Candidate peptide markers that could not be confirmed using this initial search, were reanalysed with an error-tolerant search strategy, enabling the identification of novel sequence variants. The same database was used as during the initial search, but with different parameter settings: cleavage sites fully specific C-term R and K; 2 missed cleavages allowed; mass changes - 3 common, 1 rare (all sequence variants allowed); oxidation on K, M, and P; deamidation on N and Q; false discovery rate (FDR) 2%. All possible sequence variants and their corresponding masses were recorded for peptide markers that were not identified previously.

To identify other bone proteins, and to confirm the authenticity of the samples, the MS/MS data was searched against the proteomes of *Bos taurus* (UP000009136), *Ovis aries* (UP000002356), *Vicugna pacos* (UP000504605), *Odocoileus virginianus texanus* (UP000504613), *Cervus elaphus hippelaphus* (UP000242450), *Equus caballus* (UP000002281), *Sus scrofa* (UP000008227), *Canis lupus familiaris* (UP000805418), *Vulpes vulpes* (UP0002805418), *Felis catus* (UP000011712), *Panthera leo* (UP000694399), *Lynx canadensis* (UP000472241), *Enhydra lutris kenyoni* (UP000248482), *Castor canadensis* (UP000694852), *Cavia porcellus* (UP000005447), and all sequence data available in Swissprot. The parameter settings used were: cleavage sites fully specific C-term R and K; 3 missed cleavages allowed; mass changes - 2 common, 1 rare; oxidation on K, M, and P; deamidation on N and Q; pyro-Glu on N-term glutamic acid (E) and Q; ammonia-loss on N-term cysteine (C); false discovery rate (FDR) 2%.

Finally, the results of the three searches were used to create a new database for final biomarker confirmation. This database consists of the COL1α1 and COL1α2 sequences of the initial database, all sequence variants identified in the error-tolerant search, the proteins identified in the proteome search, and common contaminants. The MS/MS data was searched against this database using the same parameter settings as the initial search. Candidate peptide markers were considered confirmed when they had at least 3 peptide spectral matches with PEP2D scores <0.01 and good coverage of the b- and y-ion series.

**Results**

Table S1: Peptide sequences corresponding to ZooMS peptide markers. The naming of peptide markers follows Brown et al. [(2021)](https://paperpile.com/c/QSVZxO/mikA/?noauthor=1). Bolded and underlined amino acids represent variations in the sequence. Markers in parentheses represent the same marker with an additional oxidation. Isoleucine and leucine are indistinguishable with MS because they have the same monoisotopic mass. Therefore, in instances where both amino acids were matched at a given sequence position, we report the residue here as isoleucine.

| **Peptide marker** | **Sequence** | **Mass** |
| --- | --- | --- |
| COL1α1 508-519 (P1) | GVQGPPGPAGPR | 1105 |
| COL1α2 978-990 (A) | **T**G**QA**G**A**VGPAG**I**R | 1154 (1170) |
|  | **S**G**HP**G**A**VGPAG**V**R | 1161 (1177) |
|  | **S**G**HP**G**S**VGPAG**V**R | 1177 (1193) |
|  | **T**G**QP**G**A**VGPAG**I**R | 1180 (1196) |
|  | **S**G**QP**G**T**VGPAG**V**R | 1182 (1198) |
|  | **I**G**QP**G**A**VGPAG**I**R | 1192 (1208) |
|  | **S**G**HP**G**T**VGPAG**I**R | 1205 (1221) |
|  | **T**G**HP**G**S**VGPAG**V**R | 1207 (1223) |
|  | **T**G**QP**G**T**VGPAG**I**R | 1210 (1226) |
| COL1α2 375-386 | EGP**A**GLPGI**D**GR | 1154 (1170) |
|  | EGP**V**GLPGI**S**GR | 1182 (1198) |
|  | EGP**M**GLPGI**D**GR | 1211 (1230) |
| COL1α2 449 | G**L**PG**S**PGN**V**GPAGK | 1239 |
|  | G**F**PG**A**PGN**V**GPAGK | 1257 |
|  | G**F**PG**A**PGN**I**GPAGK | 1271 |
|  | G**F**PG**S**PGN**V**GPAGK | 1273 |
|  | G**F**PG**S**PGN**I**GPAGK | 1287 |
|  | G**F**PG**S**PGN**V**GPAGKEGP**V**GLPGIDGRPGPIGPAG**A**R | 3311^a^ |
|  | G**L**PG**S**PGN**V**GPAGKEGP**V**GLPGIDGRPGPIGPAG**T**R | 3322^a^ |
|  | G**F**PG**S**PGN**I**GPAGKEGP**V**GLPGIDGRPGPIGPAG**A**R | 3324 (3341)^a^ |
|  | G**F**PG**A**PGN**V**GPAGKEGP**M**GLPGIDGRPGPIGPAG**A**R | 3342 (3358)^a^ |
| COL1α2 484-498 (B) | GIPGEFGLPGPAG**A**R | 1427 |
|  | GIPGEFGLPGPAG**P**R | 1453 |
| COL1α2 502-519 (C) | GPPGESGA**A**GP**A**GPIG**S**R | 1550 |
|  | GPPGESGA**A**GP**S**GPIG**S**R | 1566 |
|  | GPPGESGA**A**GP**T**GPIG**S**R | 1580 |
|  | GPPGESGA**A**GP**S**GPIG**N**R | 1593 |
|  | GPPGESGA**V**GP**S**GPIG**S**R | 1594 |
| COL1α2 889-906 | GEPG**PA**G**A**VGP**A**GA**V**GPR | 1532 |
|  | GEPG**PA**G**S**VGP**A**GA**V**GPR | 1548 |
|  | GEPG**PV**G**A**VGP**A**GA**V**GPR | 1560 |
|  | GEPG**AA**G**S**VGP**T**GA**I**GPR | 1566 |
|  | GEPG**PA**G**S**VGP**V**GA**V**GPR | 1576 |
|  | GEPG**PA**G**V**VGP**V**GA**V**GPR | 1588 |
|  | GEPG**PV**G**S**VGP**V**GA**V**GPR | 1604 |
| COL1α2 292-309 (P2) | G**PN**GE**A**G**SA**GP**S**GPPGLR | 1593 (1609) |
|  | G**ST**GE**P**G**SA**GP**A**GPPGLR | 1596 |
|  | G**PT**GE**V**G**SP**GP**A**GPPGLR | 1634 |
|  | G**SN**GE**V**G**SA**GP**P**GPPGLR | 1637 |
|  | G**PN**GE**V**G**SA**GP**P**GPPGLR | 1647 |
|  | G**ST**GE**I**G**PA**GP**P**GPPGLR | 1648 |
|  | G**PN**GE**P**G**ST**GP**A**GPPGLR | 1649 |
| COL1α2 793-813 (D) | GLPG**VA**G**AL**GEPGPLGI**A**GPPGAR | 2129 |
|  | GLPG**VA**G**SV**GEPGPLGI**A**GPPGAR | 2131^b^ |
|  | GLPG**VA**G**AV**GEPGPLGI**S**GPPGAR | 2131^c^ |
|  | GLPG**IA**G**AL**GEPGPLGI**A**GPPGAR | 2143 (2159) |
|  | GLPG**VA**G**SL**GEPGPLGI**A**GPPGAR | 2145 |
|  | GLPG**VS**G**SV**GEPGPLGI**A**GPPGAR | 2147 |
|  | GLPG**VS**G**SV**GEPGPLGI**S**GPPGAR | 2163 |
| COL1α2 454-483 (E) | GEQGPAGPPGFQGLPGP**A**G**TA**GE**A**GKPGER | 2792 |
|  | GEQGPAGPPGFQGLPGP**S**G**TA**GE**A**GKPGER | 2808 |
|  | GEQGPAGPPGFQGLPGP**A**G**TA**GE**V**GKPGER | 2820 |
|  | GEQGPAGPPGFQGLPGP**S**G**PT**GE**V**GKPGER | 2862 |
| COL1α1 586-618 (F) | GLTGPIGPPGPAGAPGDKGE**A**GPSGPAGPTGAR | 2853 (2869) |
|  | GLTGPIGPPGPAGAPGDKGE**S**GPSGPAGPTGAR | 2869 (2885) |
|  | GLTGPIGPPGPAGAPGDKGE**T**GPSGPAGPTGAR | 2883 (2899) |
| COL1α2 757-789 (G) | GPSGEPGTAGPPGTPGPQGILG**A**PG**I**LGLPGSR | 2983 (2999) |
|  | GPSGEPGTAGPPGTPGPQGILG**A**PG**F**LGLPGSR | 3017 (3033) |
|  | GPSGEPGTAGPPGTPGPQGILG**P**PG**F**LGLPGSR | 3043 (3059) |

^a^Peptide with missed cleavage.

^b^Sequence for antilocaprids (*Antilocapra americana*), bovids (*Oreamos americanus*, *Ovis canadensis*, and *Bootherium bombifrons*), cervids (*Odocoileus virginianus*, *O. hemionus*, *O. lucasi*, and *Cervalces scotti*), and canids (*Urocyon cinereoargenteus*, *Vulpes velox*, and *Canis latrans*).

^c^Sequence for camelids (*Camelops* sp. and *Hemiauchenia* sp.).
